# The relationship between attachment to pets and mental health: the shared link via attachment to humans

**DOI:** 10.1186/s12888-022-04199-1

**Published:** 2022-09-03

**Authors:** Johanna Lass-Hennemann, Sarah K. Schäfer, M. Roxanne Sopp, Tanja Michael

**Affiliations:** 1 Department of Psychology, Saarland University, Building A1 3, 66123 Saarbrucken, Germany; 2 Leibniz Institute for Resilience Research, Wallstrasse 7, 55122 Mainz, Germany

**Keywords:** Pet, Dog, Animal, Mental health, Distress, Attachment

## Abstract

**Background:**

Several studies have investigated the relationship between emotional attachment to pets and mental health with the majority of studies finding a negative relationship between emotional attachment to pets and mental health. Interestingly, attachment to pets differs from attachment to humans with studies showing that humans with an insecure attachment style form a particularly strong emotional attachment to their companion animals. Human attachment style is also related to mental health with secure attachment being associated with superior mental health. Building on those findings, the current study aimed at exploring the role of attachment to humans in the relationship between emotional attachment to pets and mental health.

**Methods:**

In this cross-sectional online survey (*N* = 610) we assessed the strength of emotional attachment to pets and attachment to humans. We further collected pet specific data as well as mental health burden in a sample of German dog owners (*M*_*age*_=33.12; 92.79% women). We used a mediation model estimating the indirect link between emotional attachment to pets and mental health burden via human attachment and the direct link between emotional attachment to pets and mental health burden simultaneously.

**Results:**

We found that attachment to humans fully mediated the positive association between emotional attachment to pets and mental health burden. A stronger emotional attachment to one’s dog was associated with lower comfort with depending on or trusting in others, whereby lower comfort with depending on or trusting in others was related to higher mental health burden. Moreover, a stronger attachment to one’s dog was also related to a greater fear of being rejected and unloved (Anxiety), which was, in turn, associated with a higher mental health burden.

**Conclusion:**

Our findings suggest that the positive link between emotional attachment to pets and mental health burden is fully accounted for by its shared variance with insecure attachment to humans in a sample mostly comprising self-identified women. Future studies need to examine whether strong emotional bonds with pets may evolve as a compensatory strategy to buffer difficult childhood bonding experiences.

## Background

Pet ownership has been linked to better mental health and enhanced well-being in the general population as well as in patients with physical and mental disorders (for a review see Wells [1]). For instance, pet ownership has been shown to be associated with lower levels of loneliness and depression and better perceived general health in older populations [2]. However, at the same time, some studies also find null or even negative effects of pet ownership on physical and mental health, raising the question whether pet ownership is robustly associated with better health (for a critical review see Herzog [3]).

To account for these diverging findings, it has been proposed that emotional attachment to pets moderates the relationship between pet ownership and mental health [4], with (mental) health-benefitting effects of pet ownership only be present in those pet owners who show a strong emotional attachment to their pets. Research suggests that humans are often strongly attached to their pets [5, 6] and sometimes report even stronger attachment to pets than to human family members [7]. Thus, recent research increasingly focused on the relationship between emotional attachment to pets and mental health. However, the current evidence does not confirm the hypothesis that a strong emotional attachment to pets is associated with better mental health. While some studies found a positive relationship between strong emotional attachment to pets and mental health [8–11], others did not find such an association [12–15], and the majority of studies even found a negative relationship between emotional attachment to pets and mental health [6, 16–20], that is, a stronger emotional attachment to one’s pet was linked to worse mental health. This effect is somewhat surprising and strongly contradicts the notion that only those pet owners who show a strong emotional attachment to pets experience (mental) health-benefitting effects of pet ownership. At the same time, the negative relationship between emotional attachment to pets and mental health raises the question what may account for this - at the first sight - paradox link.

An emerging line of research linking emotional attachment to pets to human interpersonal relationship and bonding experiences may provide first insights, with one study finding a stronger emotional attachment to pets being associated with (mostly interpersonal) childhood trauma and elevated levels of dissociation [21–23]. Other studies assessing human social support and emotional attachment to pets find that pet owners that are highly attached to their pet report lower levels of human social support [13, 24]. Furthermore, studies assessing attachment to pets, human social support mental health in the same sample found that pet owners with low levels of social support and a strong emotional attachment to pets report higher scores of loneliness and depression ([20, 25, 26]; but see [27]).

Interestingly, a person’s attachment to other humans is also related to well-being and mental health with insecure attachment styles being associated with poorer mental health (for a review see Dozier et al. [28]). Attachment theory was first proposed by Bowlby [29] to explain how infants form attachment to their primary caregivers. Ainsworth et al. [30] refined attachment theory by identifying four distinct attachment styles: (1) secure, (2) anxious, (3) avoidant, and (4) disorganized with the latter three representing insecure attachment styles. Children typically develop secure attachment styles when their primary caregiver is attentive and responsive to the children’s distress. Children with inconsistent or slow-to-respond caregivers typically develop anxious attachment styles. Avoidant attachment results from caregivers who consistently reject the infant, and disorganized attachment is believed to be an outcome of abuse and trauma [31–33]. Bowlby [33] assumed that the attachment a child forms with his or her primary caregiver early in life creates internal working models for close relationships in later life. Indeed, research shows that attachment style is relatively stable across the life span [34].

So far, little is known on the relationship between emotional attachment to pets and human attachment styles. Beck and Madresh [7] observed that emotional attachment to pets is generally rated as more secure than attachment to significant others. Taggart [35] found that people with a fearful attachment style report a stronger emotional attachment to their pets as compared to people with a secure attachment style. Furthermore, Beetz, Julius et al. [36] showed that children with an insecure-avoidant or disorganized attachment style profit more from the presence of a therapy-dog than from the presence of a friendly human under social stress.

When reviewing the findings on the association between attachment to other humans and emotional attachment to pets it has to be taken into account that while research into human attachment either assigns individuals to categories of attachment style or measures the strength of various categories of attachment (e.g., anxious or avoidant attachment) [37], most research into emotional attachment to pets comprises a unidimensional strength-based approaches ranging from weak to strong attachment. Recently, some studies have also employed categorical approaches (i.e., assessing attachment styles) to assess attachment to pets [38]. The present state of research does not allow for a conclusion which approach is better suited for research into emotional attachment to pets, however, the strength-based approach has been used more often resulting in a larger number of well-established psychometrically valid assessments [39–42].

It is yet not clear what links attachment to humans and emotional attachment to one’s pet. One pathway may lie in the use of a close emotional bond to pets as a compensatory attachment strategy for people who were not able to establish secure relationships to other people during childhood. Indirect support for this hypothesis may come from studies linking childhood trauma and childhood neglect to a stronger emotional attachment to pets [21–23] and from research showing that pet owners with low levels of social support and a strong emotional attachment to pets report higher scores of loneliness and depression ([20, 25, 26]; but see [27]).

However, to the best of our knowledge, so far there is only one dissertation project [12] that measured emotional attachment to pets and human attachment styles, as well as their levels of anxiety and depression in a sample of 300 dog, cat and horse owners. The dissertation found insecure attachment to humans to be associated with anxiety and depression. In contrast to previous findings, attachment to pets was not significantly correlated with insecure attachment to humans (anxiety and avoidance dimensions), depression, or anxiety. However, the findings should be interpreted with caution, as the study has not been peer-reviewed and assessed a relatively small and heterogenous sample including horse owners.

Given the strong bivariate relationships between emotional attachment to pets, attachment to humans and mental health, it is of great interest to assess all three measures in a relatively homogeneous sample of dog owners. The relationship between insecure attachment to humans and poor mental health has been very consistently shown. The association between attachment to pets and poor mental health has been less extensively researched, however, the majority of studies suggests that a stronger emotional attachment to pets is linked to worse mental health [6, 16–20]. Building on evidence that provided support for more insecure and anxious attachment to humans being related to a stronger emotional attachment to pets, we assumed that the relationship between emotional attachment to pets and mental health might be accounted for their shared variance with attachment to humans.

Thus, the present cross-sectional survey examined attachment to both pets and humans, mental health, and several pet specific variables in a sample of 610 German dog owners. We hypothesized that (i) a stronger emotional attachment to one’s dog is associated with higher mental health burden, and (ii) a stronger emotional attachment to one’s dog shows a negative association with indicators of secure human attachment and a positive association with indicators of insecure human attachment. Moreover, to disentangle the link between emotional attachment to pets and mental health burden and the association between emotional attachment to pets and human attachment and their respective associations with mental health burden, we used a mediation model comprising a link to mental health via (dimensional) human attachment (indirect paths) and direct path from emotional attachment to pets to mental health. We assume that only the indirect paths account for the link between emotional attachment to pets and mental health burden and thus, insecure human attachment is the key to explain higher mental health burden related to stronger emotional attachment to pets.

## Methods

### Sample recruitment and

Respondents were recruited online by distributing the link to the survey on special webpages for dog owners and social media (i.e., Facebook groups, websites of dog schools). Sample recruitment for the 20-minute online survey took place between June and August 2019. All respondents gave written informed consent in accordance with the Declaration of Helsinki and its latest amendments [43].

### Sample characteristics

The mean age of the 610 respondents was 33.12 years (*SD* = 11.88, age range: 18–73 years). Five-hundred-sixty-six respondents (92.79%) identified as women, 7.05% identified as men and one respondent (0.16%) identified as non-binary. Twenty-nine respondents (4.75%) reported to have eight years of school, 25.25% attended school for 10 years, 43.61% reported to have 11 to 13 years of school, and 22.13% had finished university, while another 4.26% were students or indicated to have a different educational level. One-hundred-ninety-seven respondents (32.95%) were married or lived in a registered civil partnership, 0.65% were widowed, 2.46% were divorced, 41.64% lived in a partnership and 22.30% indicated to be single. The majority of the sample (67.05%) reported to own one dog, 24.92% owned two dogs, 4.43% owned three dogs, and 3.61% reported to have more than three dogs (for details on dog ownership see Table 1).

**Table 1:** Characteristics of dog ownership in the study sample

**Characteristics**	
Number of dogs (n/%)	
One dog	409 (67.05)
Two dogs	152 (24.92)
Three dogs	27 (4.43)
More than three dogs	22 (3.61)
Dog age	52.47 months (43.20) 4.37 years (3.60)
Sex dog	52.13% female
Neutering (n/%)	
Yes	280 (45.90)
No	329 (53.93)
Duration of dog ownership	44.74 months (37.88) 3.73 years (3.15)
Average time spent with the dog per day	163.07 min (88.97)
Origin of the dog (n/%)	
Breeder	295 (48.36)
Animal welfare	150 (24.59)
Internet	76 (12.46)
Other	89 (14.59)

### Materials and measures

#### Socio-demographic data and dog-related information

The online form started with several questions assessing socio-demographic information (i.e., gender, age) and dog-related information (i.e., age of dog and duration of ownership, sex and neutering status, origin of the dog [e.g., breeder, animal welfare]) and average daily time spent with the dog. Subsequently, respondents answered three standardized questionnaires regarding their attachment to other people, and to their dog and their mental health burden.

#### Lexington Attachment to Pets Scale (LAPS)

We employed the German translation of the LAPS [39, 41] to assess emotional attachment to one’s pet. The scale can be used for cats and dog owners and consists of 23 items (for example “My pet understands me.”, “My pet and I have a very close relationship.”), which are rated on a 4-point Likert scale. Higher scores indicate a stronger attachment to pet. In the current sample internal consistency was high, as reflected in Cronbach’s alpha (α) = 0.89, and McDonald’s omega (ω) = 0.89.

#### Revised Adult Attachment Scale (R-AAS)

We employed the German translation of the R-AAS [44] to assess attachment to humans in our study. The R-AAS consists of three subscales: comfort with emotional closeness (Closeness), comfort with depending on or trusting in others (Dependence), and anxious concern about being abandoned or unloved (Anxiety). Participants are asked to respond in terms of their general orientation towards close relationships. The R-AAS consists of 18 items that are answered on a 5-point Likert scale, with higher scores reflecting stronger closeness, dependence, or anxiety. Closeness and Dependence were seen as an (inverse) measure of avoidant attachment from other measures and Anxiety may be viewed in line with anxious attachment of other attachment scales [45]. In the current study, all subscales demonstrated sufficient internal consistency reflected in α = 0.76 and ω = 0.77 for Closeness, α/ω = 0.86 for Dependence, and α/ω = 0.90 for Anxious Attachment, respectively.

#### Brief Symptom Inventory (BSI)

The current burden on mental health was assessed using the German version of the Brief Symptom Inventory [46]. The BSI is a 53-item self-report measure that assesses symptomatic distress using nine subscales. For the purpose of the current study only the global severity index (GSI) was used to indicate mental health burden. In the present sample, the GSI showed very good internal consistency, α/ω = 0.97.

### Data collection and analysis

All measures were collected via the online platform SoSci Survey [47]. Analyses were conducted using RStudio version 2021.09.1 [48] and the *lavaan* package [49] for structural equation modeling. Descriptive statistics were computed to illustrate sample characteristics in terms of frequencies, means (*M*), and standard deviations (*SD*). Pearson correlations were computed to assess the relationship between variables included in our models. We used mediation models based on structural equation modeling to shed light on the relationship between emotional attachment to dogs and mental health burden and to examine if their link may lie in their shared association with human attachment. To note, our model does not imply that emotional attachment to pets causally results in human attachment. Models are solely based on cross-sectional data and use the mediation model to explore and visualize the relationship between emotional attachment to pets and mental health burden. For that purpose, the subscales of the AAS-R were used as parallel mediators and indirect effects were estimated as product terms. All models were adjusted for age, gender, and educational level. The effects were estimated based on 5,000 bootstrap samples using a bias-corrected percentile method. All coefficient estimates were standardized, with a *p*-value < 0.05 indicating statistical significance. Post-hoc power calculations for indirect effects were performed according to the procedure described in Schoemann et al. [50] based on Monte Carlo simulations. Data and analytic code for all analyses are available at https://osf.io/w8u67/?view_only=8861b2aca94544a2a5b482c33e4232d6 [51].

## Results

### Bivariate relationships

Table 2 presents the bivariate relationships between study variables. Attachment to one’s dogs is negatively related to comfort with emotional closeness (Closeness), *r* = − .10, *p* = .013, and comfort with depending on or trusting others (Dependence), *r* = − .16, *p* < .001, which reflect inverse measures of avoidant attachment as assessed in other attachment scales. Anxious attachment is positively associated with attachment to one’s dogs, *r* = .18, *p* < .001. Moreover, there is a positive relationship between attachment to one’s dog and mental health burden, *r* = .16, *p* < .001. The attachment dimensions of Closeness, *r* = − .39, *p* < .001, and Dependence, *r* = − .51, *p* < .001, were negatively associated with mental health burden, while anxious attachment, *r* = .56, *p* < .001, was positively correlated with mental health burden.

**Table 2:** Bivariate relationship between study variables

	*M* (*SD*)	1.	2.	3.	4.	5.	6.	7.
1. Age	33.12 (11.89)	—						
2. Educational level	6.04 (1.73)	− 0.15**	—					
3. Attachment to dog	56.01 (8.39)	− 0.22**	− 0.15**	—				
4. Attachment - Closeness	20.10 (4.61)	0.06	0.10**	− 0.10*	—			
5. Attachment - Dependence	19.73 (5.36)	0.08	− 0.18**	− 0.16**	0.65**	—		
6. Attachment – Anxiety	15.90 (6.20)	− 0.31**	− 0.07	0.17**	− 0.44**	− 0.68**	—	
7. Global mental health burden (GSI)	56.89 (14.05)	− 0.24**	− 0.11*	0.16**	− 0.39**	− 0.50**	0.56**	—

*Note*. Means and standard deviations of sum scores and *T* scores in case of mental health burden

* *p* < .05

** *p* < .01

### Mediation model

**Fig. 1:**
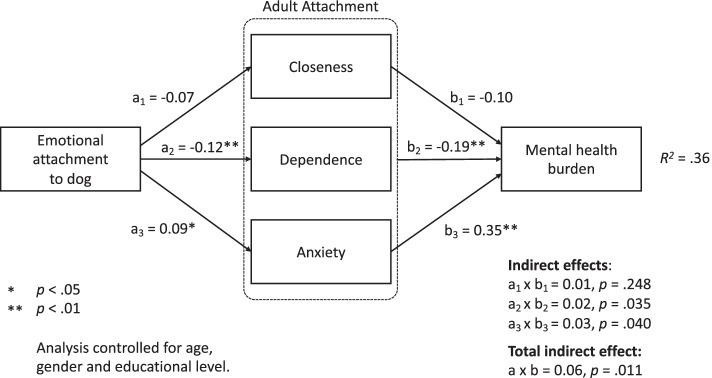
Full mediation model

To shed light on the cross-sectional relationships between our study variables, we examined attachment to others (i.e., Closeness, Dependence, Anxiety) as mediators of the relationship between emotional attachment to dog and mental health burden. First, we compared a model including a direct effect from emotional attachment to dog to mental health burden with a model only including indirect paths via attachment to others. As these models did not reveal a significant difference in fit, ∆χ^2^(1) = 1.48, *p* = .279, we used the more constrained model only including indirect paths. In this model that was adjusted for age, gender and educational level, we found a significant total indirect effect that fully mediated the relationship between emotional attachment to dog and mental health burden, a x b = 0.06, 95% CI [0.01, 0.11], *p* = .011. This effect was based on a significant indirect effect via Dependence, a_2_ x b_2_ = 0.02, 95% CI [0.01, 0.05], *p* = .035, and Anxiety, a_3_ x b_3_ = 0.03, 95% CI [0.01, 0.07], *p* = .040, but not via Closeness, a_1_ x b_1_ = 0.01, 95% CI [-0.01, 0.03], *p* = .278 (see Fig. 1). A stronger emotional attachment to one’s dog was associated with lower comfort with depending on or trusting in others (Dependence), whereby lower comfort with depending on or trusting in others was related to higher mental health burden. Moreover, a stronger emotional attachment to one’s dog was also related to a greater fear of being rejected and unloved (Anxiety), which was in turn associated with higher mental health burden.

### Post-hoc power calculation

Given the current sample size and the bivariate associations of included variables, statistical power was sufficient for indirect effects via Dependence, 1 - β = 0.81, and Anxiety, 1 - β = 0.98, but low for an indirect effect via Closeness, 1 - β = 0.33.

## Discussion

In the present study we assessed emotional attachment to one’s pets and attachment to humans as well as mental health burden in a large sample of dog owners. A stronger emotional attachment to one’s dog was associated with a higher mental health burden. In line with our expectations, we found that attachment to humans fully mediated this relationship. A stronger emotional attachment to one’s dog was associated with lower comfort with depending on or trusting in others (Dependence), and a lower comfort with depending on or trusting in others was related to higher mental health burden. Moreover, a stronger emotional attachment to one’s dog was also related to a greater fear of being rejected and unloved (Anxiety), which was, in turn, associated with higher mental health burden. Thereby, our study provides evidence that the positive association between emotional attachment to one’s dog and mental health burden, which might be surprising at the first sight, may reflect an association driven by their shared association with human attachment. Contrary to our expectations we did not find a mediating effect for the Closeness dimension of the R-AAS, even though the bivariate relationships between Closeness and emotional attachment to one’s dog and Closeness and mental health burden were significant.

In our study, we were able to replicate the findings that insecure attachment to humans is related to stronger emotional attachment to pets. These findings are in line with research showing that people rate the emotional attachment to their pets as generally more secure than the attachment to significant others [7], and studies showing that insecurely attached children benefit more from the presence of a therapy-dog than of a friendly human under social stress [52]. Thus, from a developmental perspective the link between an insecure attachment to humans and stronger emotional attachment to pets might reflect a compensatory attachment strategy for people who were not able to establish secure relationships to other people during childhood. Those people may build more close relationships with pets that might be perceived as more reliable and less threatening. In accordance with our speculation Julius et al. [53] showed that in a sample of 160 children who had experienced abuse, neglect, or traumatic loss, reports of a secure attachment to a pet, especially a dog or cat, were four times more likely than a secure attachment to their human caregiver. Furthermore, female college students with self-reported neglect during childhood reported a stronger attachment to companion animals as compared to college students without self-reported neglect [21]. Furthermore, there is some research on the relationship between attachment to pets and dissociation. High levels of dissociation are strongly correlated with previous traumatic experiences like sexual or physical abuse [54]. As dissociation also correlates with attachment to pets [22, 23], Brown and colleagues have hypothesized that a subset of people highly attached to companion animals has a history of abuse or trauma. Future prospective studies should investigate whether a high attachment to pets develops as a response to negative human bonding experiences and interpersonal trauma, and whether this compensatory attachment strategy might harm or benefit psychological well-being and mental health. Such studies need to include respondents with diverse bonding experiences during childhood as well as respondents with and without pets.

In the current study, attachment to pets was positively associated with mental health burden. These results are in line with the majority of previous studies showing a positive association between emotional attachment to pets and mental health burden. However, there are also some studies relating stronger emotional attachment to pets to better mental health. The variety of sample characteristics investigated in the different studies might account for these findings. The current study used a community-based sample without any restrictions (except of dog ownership), whereas previous studies often investigated more specific samples like older women living alone [11], students [22], or occupations at high risk of traumatization [17].

One misconception in previous research is to treat a strong emotional attachment to pets as an equivalent of a secure attachment to pets. In our study, we used the Lexington Attachment to Pets Scale [39], a well-validated and widely used measure of attachment to pets. The LAPS has been designed to assess the strength of emotional attachment to pets, but it does not provide any information about attachment style and does not correspond to the attachment dimensions employed in human attachment style questionnaires. Thus, the use of the LAPS does not allow for a conclusion on whether a strong attachment to pets corresponds to a secure attachment to pets. We decided to employ the LAPS, because it represents the most widely used and best validated questionnaire on attachment to pets. The Pet Attachment Questionnaire (PAQ) is a more recently developed questionnaire [38], which is based on Bowlby’s attachment theory and may thus better assess a secure attachment to one’s pets. However, the PAQ is less employed in companion animal research and there is (to our knowledge) no German version, which has undergone psychometric validation. Nevertheless, future research needs to provide a psychometrically valid version of the PAQ forming a base for further studies incorporating both measures and may shed light on the relationship between (more similar) indicators of emotional attachment to pets and humans. These studies should also focus on the question what degree or style of attachment to pets might relate to well-being and mental health.

### Limitations

Some limitations have to been taken into account when interpreting findings from the current study. The first and probably most important limitation reflects the fact that the current study is cross-sectional in design. Therefore, our mediation model aimed at exploring and visualizing cross-sectional associations and was not designed to suggest causal relationships. Future studies have to examine the longitudinal interplay between emotional attachment to pets, attachment to humans, mental health and well-being. These studies may start in early life phases to provide evidence on the assumption of strong attachment to pets being a compensatory strategy for insecure human attachment by examining the complex relationship between (aversive) childhood experiences and/or childhood trauma and emerging emotional attachment to pets. In our study, we only assessed dog owners. Thus, we cannot generalize our findings to owners of other types of pets. We decided to question dog owners only, because dogs as “man’s best friend” received most attention in companion animal research and in previous research dog owners reported the highest attachment to their pets [55, 56]. Nevertheless, future studies should extend these findings to other types of pets.

Furthermore, the majority of our participants self-identified as women. Thus, conclusions from the current sample remain limited to a highly selective and potentially biased convenience sample and may only apply to self-identified women who are generally more attached to pets than men (see Herzog [57] for a review). For human attachment, gender differences are not consistently found across the lifespan [58]. However, gender differences are commonly present with respect to several mental health disorders [59–61]. Thus, it would be very interesting to replicate these findings in a more diverse and gender balanced sample and in different age groups.

Finally, we did not find a mediating effect for the Closeness dimension of the R-AAS. However, post-hoc power analysis showed that the power was too low to detect an indirect effect via Closeness. Thus, the absence of a significant mediation by the Closeness dimension should not be misinterpreted as evidence for the absence of this effect as it may reflect a lack of statistical power to detect such a mediation.

## Conclusion

Our findings contribute to the growing body of evidence linking emotional attachment to pets to mental health burden. To the best of our knowledge, this study is the first to show that the positive relationship between emotional attachment to pets and mental health burden may be fully accounted for by shared variance between emotional attachment to pets and insecure attachment to humans. To further shed light on the relationship between emotional attachment to pets, human attachment, and mental health, prospective studies are needed that use more similar approaches to attachment to humans and pets, and investigate whether a stronger emotional attachment to pets develops as a response to negative bonding experiences, and whether this compensatory strategy benefits or harms psychological well-being.

## Data Availability

Data and analytic code for all analyses are available at Open Science Framework, link: https://osf.io/w8u67/?view_only=8861b2aca94544a2a5b482c33e4232d6.

## Abbreviations

BSI: Brief Symptom Inventory
GSI: Global Severity Index
LAPS: Lexington Attachment to Pets Scale
R-AAS: Revised Adult Attachment Scale
